# Effect of Esculetin on *Tert*-Butyl Hydroperoxide-Induced Oxidative Injury in Retinal Pigment Epithelial Cells In Vitro

**DOI:** 10.3390/molecules27248970

**Published:** 2022-12-16

**Authors:** Woo Kwon Jung, Su-Bin Park, Hwa Young Yu, Yong Hwan Kim, Junghyun Kim

**Affiliations:** Department of Oral Pathology, School of Dentistry, Jeonbuk National University, Jeonju 54896, Republic of Korea

**Keywords:** age-related macular degeneration, apoptosis, esculetin, oxidative stress, retinal pigment epithelial cell

## Abstract

Esculetin is a coumarin-derived compound with antioxidant and anti-inflammatory properties. The current study aims to evaluate the therapeutic implications of esculetin on retinal dysfunction and uncover the underlying mechanisms. *Tert*-butyl hydroperoxide (*t*-BHP) at a concentration of 300 μM was used to induce oxidative stress in human retinal pigment epithelial cell line (ARPE-19) cells. Esculetin at concentrations below 250 μM did not cause cytotoxicity to ARPE-19 cells. Cell viability analysis confirmed that *t*-BHP induced oxidative injury of ARPE-19 cells. However, ARPE-19 cells were protected from *t*-BHP-induced oxidative injury by esculetin in a concentration-dependent manner. As a result of the TUNEL assay to confirm apoptosis, esculetin treatment reduced the number of TUNEL-positive cells. Esculetin down-regulated the expression levels of Bax, Caspase-3, and PARP and up-regulated the expression level of Bcl2. Collectively, this study demonstrates that esculetin exerts potent antioxidant properties in ARPE-19 cells, inhibiting *t*-BHP-induced apoptosis under the regulation of apoptotic factors.

## 1. Introduction

The prevalence rate of age-related macular degeneration (AMD) is increasing significantly by age 50 worldwide [1]. The early stages of AMD are characterized by changes in the retinal pigment epithelial cells (RPEs) [2]. The dry type of AMD (dAMD) progresses slowly with geographic atrophy that causes the degeneration of RPEs in the macula [3]. Recently, the use of vascular endothelial growth factor (VEGF) antagonists to inhibit the VEGF signaling pathway has successfully diminished the development of the wet type of AMD (wAMD) in human subjects [4]. In numerous clinical trials, intravitreally injected anti-VEGF agents, including bevacizumab, ranibizumab and aflibercept, notably suppressed neovascularization and stabilized vision loss [5]. However, there is currently no cure for dAMD. The main cause of dAMD is oxidative stress. Excessive generation of reactive oxygen species (ROS) as a result of oxidative stress is associated with RPE dysfunction [6,7].

The retinal pigment epithelium is a single-layered cell located between the retinal photoreceptors and choroidal vessels [6,8,9]. RPEs play an important role in the mechanical and metabolic support of the photoreceptors [10]. In addition, the dysfunction of RPEs is a major source of some eye diseases, such as proliferative vitreous retinopathy, uveitis, and AMD [11,12]. RPEs are very vulnerable to oxygen tensions and oxidative stress during ischemia-related eye diseases [6,7]. The initial stage that occurs in dAMD is damage to RPEs by oxidative stress [13,14].

The various anti-oxidative agents have been shown to prevent retinal degeneration in experimental animals and patients with photoreceptor degeneration [15,16]. In the Age-Related Eye Disease Study (AREDS), a mixture of various antioxidants (vitamins C and E, β-carotene, and zinc), also known as an AREDS formula, reduced the risk of progression of macular degeneration by 25% [17]. Studies on many useful compounds, including α-tocopherol and β-carotene and plant antioxidants, have received a lot of attention in the food and pharmaceutical fields. Esculetin is a derivative of coumarin present in many plants, such as *Artemisia capillaris* and *Citrus limonia* leaves, used in herbal tea [18]. Esculetin exhibits various biological activities such as xanthine oxidase inhibitor [19], cancer growth inhibitor [20], and platelet aggregation inhibitor [21]. As a result of its polyphenolic structure, esculetin also exhibits an antioxidant property.

Esculetin has been traditionally used as a natural medicine and has important biological activities such as anti-cancer, anti-inflammation, and neuroprotection. Esculetin can regulate a variety of enzymes, such as cyclooxygenase, lipoxygenase, and inducible nitric oxide synthase, associated with cytoprotective properties [22]. Esculetin protects human corneal epithelial cells from oxidative damage through its scavenging of free radical properties and through the activation of Nrf2 signaling [23]. Esculetin also inhibited lipopolysaccharide-induced inflammation and cell death in retinal pigment epithelial cells [24]. In this study, we attempted to investigate the pharmaceutical role of esculetin on oxidative injury to RPEs. The study aimed to assess the antioxidant efficacy of esculetin against *t*-BHP-induced oxidative stress in human RPEs. There are few articles regarding the effects of antioxidants on retinal tissues. However, this study showed the detailed mechanism and signaling of the antioxidant effects of esculetin against oxidative stress for the first time.

## 2. Results

### 2.1. Esculetin Inhibits t-BHP-Induced Oxidative Injury of ARPE-19 Cells

**Figure 1:**
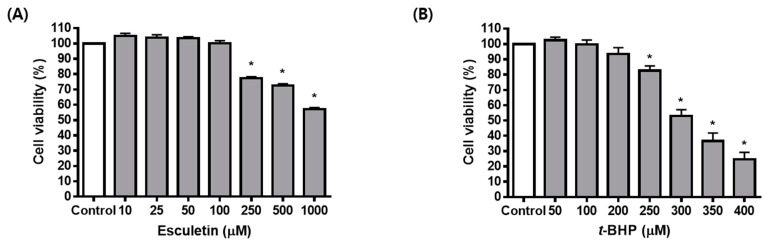

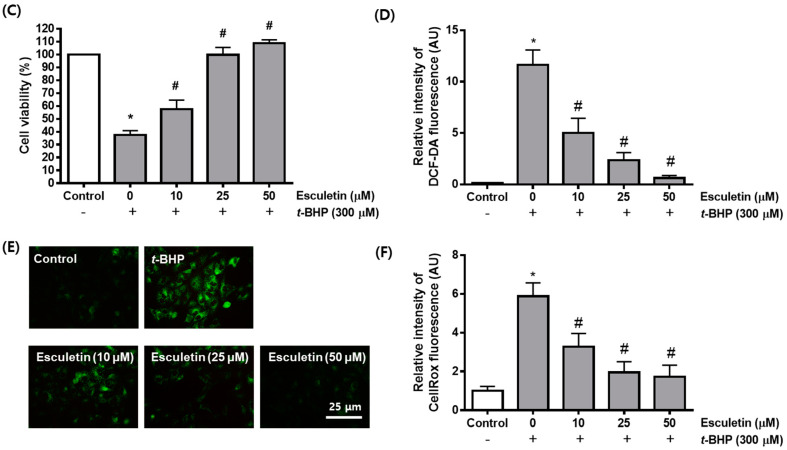
Effect of esculetin on ARPE-19 cell injury. Cell viability of ARPE-19 cells exposed to different concentrations of esculetin (**A**), *t*-BHP (**B**), and *t*-BHP with esculetin (**C**) for 24 h. (**D**) Changes in ROS levels in ARPE-19 cells exposed to *t*-BHP with esculetin for 24 h were detected using DCFH-DA dye. (**E**,**F**) The intracellular levels of ROS were measured using CellROX green reagent. Scale bar = 25 μm. The values in the bar graphs represent the means ± SEM, *n* = 5. * *p* < 0.05 vs. control group, # *p* < 0.05 vs. *t*-BHP-treated group.

Treatment for 24 h with esculetin alone confirmed the concentration that did not affect cell viability, and there was no effect up to 100 μM (Figure 1A). *t*-BHP treatment for 24 h was found to decrease cell viability in a concentration-dependent manner, with cell viability being approximately 40% at 300 μM (Figure 1B). In addition, the combination of *t*-BHP and esculetin was found to increase cell viability in a concentration-dependent manner up to 100% at 50 μM (Figure 1C). The effects of esculetin and *t*-BHP on ARPE-19 cells were also confirmed using a DCF-DA assay. It was confirmed that *t*-BHP at a concentration of 300 μM concentration promoted ROS generation in ARPE-19 cells, while esculetin inhibited it in a dose-dependent manner (Figure 1D). Similarly, the CellROX assay and its fluorescence microscopy images revealed an increase in the CellROX fluorescence signal in cells treated with *t*-BHP. However, *t*-BHP-induced total ROS level was suppressed in esculetin-treated cells (Figure 1E,F).

### 2.2. Esculetin Reduces t-BHP-Induced Apoptosis of ARPE-19 Cells

**Figure 2:**
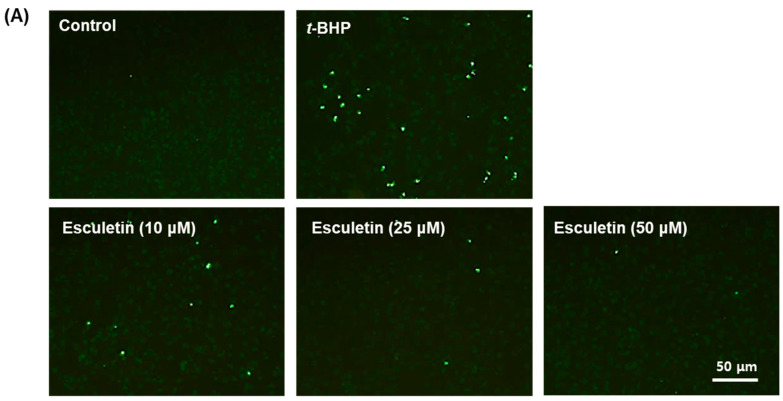

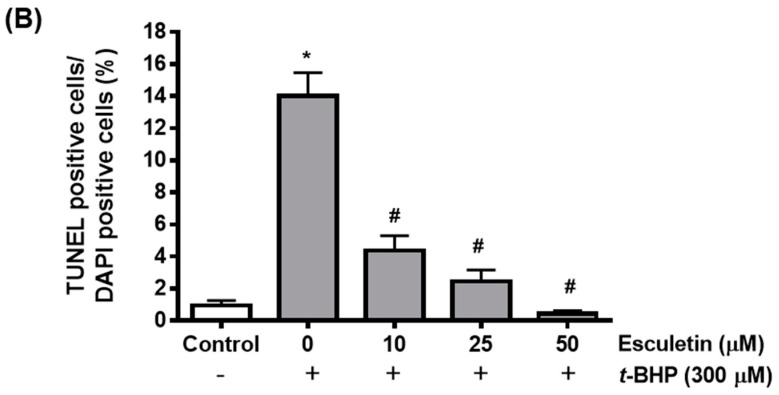
Effect of esculetin on *t*-BHP-induced apoptosis in ARPE-19 cells. (**A**) Apoptosis of ARPE-19 cells exposed to *t*-BHP with esculetin for 24 h was detected using TUNEL staining. ×100 magnification. Scale bar = 50 μm. (**B**) The values in the bar graphs represent the means ± SEM, *n* = 5. * *p* < 0.05 vs. control group, # *p* < 0.05 vs. *t*-BHP-treated group.

TUNEL staining was conducted to confirm that esculetin has a cytoprotective effect through anti-apoptotic activity. In ARPE-19 cells treated with only *t*-BHP, apoptotic cells increased 13 times compared to the untreated control group, and in cells treated with esculetin, apoptotic cells significantly decreased in a concentration-dependent manner, with apoptosis being approximately 1% at 50 μM esculetin (Figure 2).

### 2.3. Esculetin Regulates Apoptosis-Related Signaling Pathways in ARPE-19 Cells

**Figure 3:**
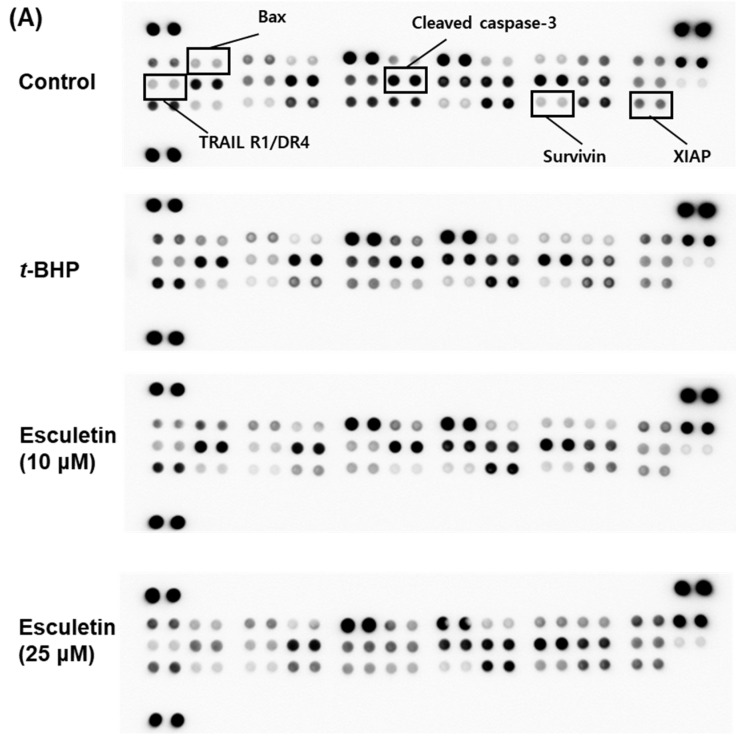

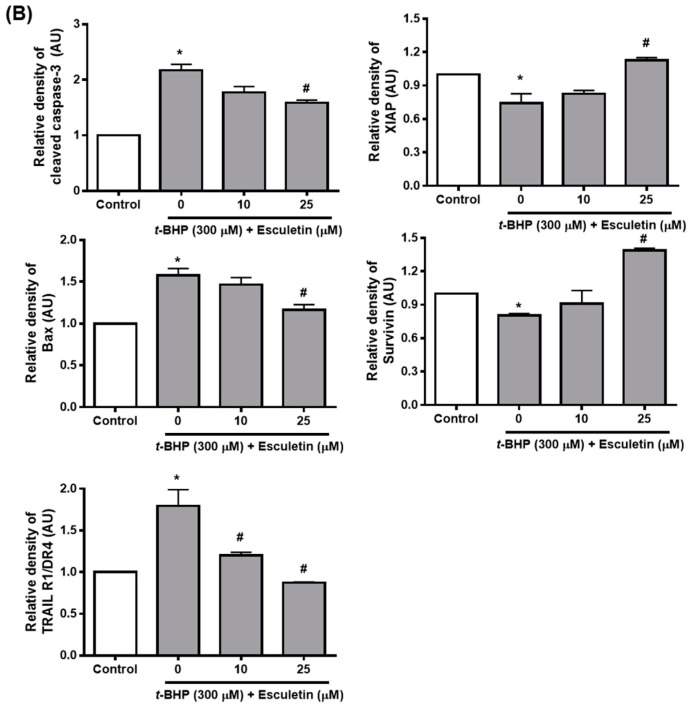
Effect of esculetin on apoptosis-related signaling pathways in ARPE-19 cells. (**A**) Apoptosis-related protein assay. (**B**) The values in the bar graphs represent the means ± SEM, *n* = 4. * *p* < 0.05 vs. control group, # *p* < 0.05 vs. *t*-BHP treated group.

We performed a human apoptosis array to determine the effect of esculetin on the expression of pro-apoptotic and anti-apoptotic proteins, as shown in Figure 3. The pro-apoptotic factors, such as cleaved caspase-3, Bax, and TRAIL R1/R4, were significantly down-regulated to almost the levels of the control group in esculetin-treated ARPE-19 cells. The expression of anti-apoptotic proteins, such as XIAP and survivin, was increased by 20% above the levels of the control group in ARPE-19 cells treated with esculetin.

### 2.4. Esculetin Regulates the Expression of Apoptosis-Related Proteins in ARPE-19 Cells

**Figure 4:**
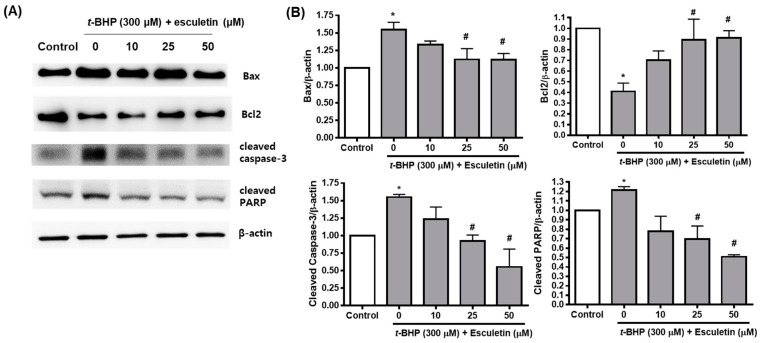
Effect of esculetin on the expression of apoptosis-related proteins in ARPE-19 cells. (**A**) The protein expression levels of Bax, bcl-2, cleaved caspase-3, and cleaved PARP. (**B**) The values in the bar graphs represent the means ± SEM, *n* = 4. * *p* < 0.05 vs. control group, # *p* < 0.05 vs. *t*-BHP treated group.

The effects of *t*-BHP and esculetin on ARPE-19 cells were also confirmed at the protein level. *t*-BHP increased and decreased the protein expression of pro-apoptosis markers (Bax, cleaved caspase-3 and cleaved PARP) and anti-apoptosis marker (Bcl-2), respectively. Esculetin was confirmed to restore the protein expression of apoptosis markers in a dose-dependent manner to almost the levels of the control group (Figure 4).

### 2.5. Esculetin Regulates the Expression of Apoptosis-Related mRNA in ARPE-19 Cells

**Figure 5:**
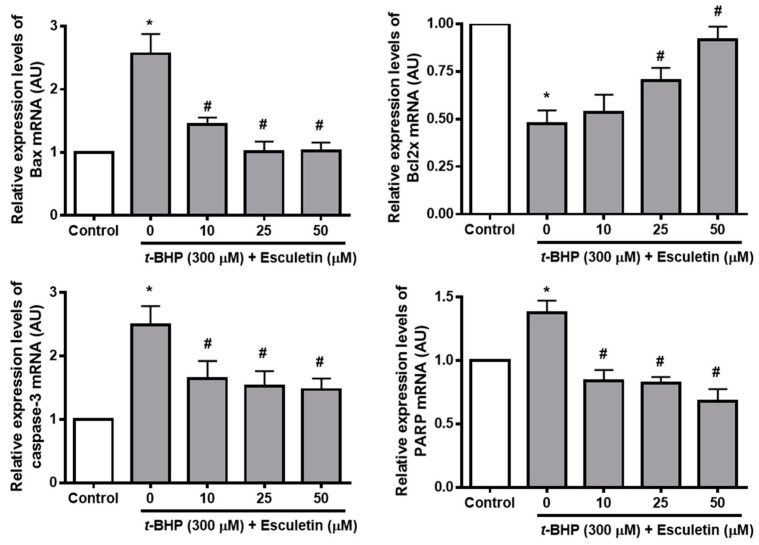
Effect of esculetin on the expression of apoptosis-related mRNA in ARPE-19 cells. The levels of mRNA expression of Bax, bcl-2, caspase-3 and PARP. The values in the bar graphs represent the means ± SEM, *n* = 4. * *p* < 0.05 vs. control group, # *p* < 0.05 vs. *t*-BHP treated group.

For further identification of the mechanism of esculetin treatment inhibiting ARPE-19 cell apoptosis and promoting viability, we also performed RT-qPCR to detect mRNA levels of apoptosis-related factors such as Bax, Bcl-2, caspase 3, and PARP. *t*-BHP increased and decreased the mRNA levels of pro-apoptosis markers (Bax, PARP, and Caspase-3) and anti-apoptosis markers (Bcl-2), respectively. Esculetin was confirmed to restore the mRNA expression of apoptosis markers in a dose-dependent manner to almost the levels of the control group (Figure 5).

## 3. Discussion

In the present study, we investigated the effect of esculetin on inhibiting apoptosis in ARPE-19 cells induced by oxidative stress. The loss of RPE is one of the leading causes of several eye diseases, including age-related macular degeneration. Macular degeneration is associated with major pathogenic mechanisms such as decreased cell volume, damaged DNA repair systems, increased apoptosis, and increased oxidative stress.

A typical cause of dAMD is damage to RPEs. RPEs play an important role in maintaining visual functions located between the choroid and photoreceptors [25]. The destruction of RPEs is one of the major pathological changes in dAMD. Factors that cause their destruction include oxidative stress, apoptosis, calcium overload, lack of nutrients and oxygen, and mitochondrial dysfunction [26,27,28]. The excessive generation of reactive oxygen species (ROS) in the retina elicits the degeneration of photoreceptor cells and retinal pigment epithelial cells, and this is considered a causative factor of retinal degenerative diseases. Mitochondria is an important endogenous source of ROS, while exogenous ROS is generated under various conditions, such as solar radiation and smoking [29]. The retina has the highest oxygen consumption rate [30]. In the retina, 60% of retinal mitochondria are located in photoreceptor cells [31], which may exacerbate photo-oxidative retinal degeneration [32]. There is an urgent need for effective treatment of dAMD. Increasing oxidative damage in experimental and clinical studies is related to the cause of dAMD, and therefore, a material with antioxidant effects can be a treatment method for dAMD [33].

There have been many reports of studies showing the possibility that dAMD is caused by RPE apoptosis, and antioxidants have been used as a strategy to protect against damage from RPEs. It has been reported that RPEs are avoided from ROS production and caspase-3 and -9 activation by the treatment with astaxanthin, a well-known antioxidant [34]. In addition, when used with ascorbic acid, which acts as an antioxidant, astaxanthin showed a superior antioxidant effect compared to each drug alone, demonstrating a synergistic effect [35]. Another study has demonstrated that OT-551, a disubstituted hydroxylamine with antioxidant properties, can help to maintain vision in dAMD patients [36]. Furthermore, the synergistic action of zeaxanthin and vitamin E or C emphasized the importance of antioxidant activity in effectively protecting RPEs from oxidative damage caused by photosensitive reactions [37]. Antioxidant supplementation has been proposed as a method to reduce retinal damage caused by oxidative stress [38]. There are several pieces of evidence that show that various antioxidant compounds, including carotenoids, flavonoids, zinc, and vitamins A, C, and E, protect RPEs from oxidative stress [38,39,40]. Clinically, the Age-related Eye Disease Study (AREDS) reported that dietary supplements taken with multivitamins alone or with zinc slowed the progression of dAMD in patients [17]. However, high-dose vitamin supplements can be harmful to patients with other risk factors. For example, vitamin A increases the risk of lung cancer in smokers, and zinc supplementation has the disadvantage of increasing the risk of urogenital complications [41,42]. Furthermore, vitamin E increases the risk of prostate cancer, vascular disease, and diabetic heart failure [43,44]. Therefore, a new RPE protective material is needed.

When ARPE-19 cells are exposed to *t*-BHP for several days, it appears to activate aging. Exposure to *t*-BHP has been proven to interfere with the junctional integrity of RPE and induce lipid peroxidation, endoplasmic reticulum Ca^2+^ release and increased mitochondrial inner membrane permeability [45,46,47,48,49,50,51,52]. *t*-BHP has also been shown to induce membrane leakage leading to cell death and cell lysis by oxidizing lipids in the membrane bilayer [53]. In this study, esculetin, as an antioxidant, protected ARPE-19 cells from oxidative stress caused by *t*-BHP.

Organic peroxide *t*-BHP induces oxidative stress via the peroxyl and alkoxyl radical pathways as well as the glutathione peroxidase pathway via cytochrome P450. It is generally used to investigate the effects of oxidative stress on cells and tissues. It has been used instead of H_2_O_2_ in oxidative stress studies [54,55]. In addition, *t*-BHP, a pro-oxidant compound, induces the production of free radicals via cytochrome P450 and induces the generation of OH-radicals, such as lipid peroxides. ROS also inhibits cell proliferation by promoting oxidative stress-induced apoptosis [54,56,57,58]. Esculetin has been shown to be a potent antioxidant in various cells [59].

In our study, esculetin significantly increased the survival rate in ARPE-19 cells from cell damage caused by *t*-BHP and reduced apoptosis by affecting related proteins and mRNA levels. Specifically, 50 μM of esculetin has been found to strongly protect ARPE-19 cells against oxidative damage. We evaluated the expression levels of protein and mRNA levels of pro-apoptotic as well as anti-apoptotic factors to confirm the apoptosis inhibitory effect of esculetin in ARPE-19 cells. There is a down-regulation of Bax, cleaved caspase-3, and cleaved PARP in esculetin-treated ARPE-19 cells. Anti-apoptotic Bcl2 protein and mRNA levels increased in a concentration-dependent manner with esculetin treatment. Esculetin has a potent antioxidant property that inhibits apoptosis by affecting apoptosis-related factors in ARPE-19 cells. Consistently, esculetin has been shown to have antioxidative activity in many previous reports. Esculetin reduces oxidative stress by inhibiting neutrophil-dependent superoxide anion production and lipid peroxidation and by scavenging free radicals [60,61,62,63]. In rat liver tissue, esculetin has been shown to reduce the incidence of liver lesions, including hepatocellular edema, leukocyte infiltration, and necrosis induced by *t*-BHP [63]. In addition, esculetin protected H_2_O_2_-induced lipid peroxidation, protein carbonyl and DNA damage in hamster lung fibroblast cells (V79-4) [64]. In oxidation-induced H9c2 cells, after esculetin treatment, Bcl-2 expression is up-regulated and Bax expression down-regulated, and it inhibits the activity of caspase-3. As a result, esculetin improved viability in hypoxia/reoxygenation-stimulated H9c2 cells, suppressed oxidative stress, and inhibited cell death [65]. Furthermore, esculetin exerts anti-apoptosis activity in the mouse model of middle cerebral artery occlusion by up-regulating Bcl-2 expression and down-regulating Bax expression and downstream cleaving caspase-3 [66]. Based on these previous results, we also showed the antioxidant effect of esculetin in ARPE-19 cells.

In the present study, the antioxidative and anti-apoptotic activities of esculetin against human RPEs were evaluated. However, these activities of esculetin were not compared with other well-known antioxidants in vitro, thereby serving as a study limitation. The in vivo experiment was required to confirm the effectiveness of esculetin in the animal model for dAMD, which served as another limitation of the study. Therefore, the detailed beneficial role of esculetin in dAMD requires further studies.

In conclusion, esculetin can protect RPEs from oxidative stress-induced apoptosis and has the potential to act as a treatment for eye diseases, including dAMD.

## 4. Materials and Methods

### 4.1. Cell Culture

Human adult pigment epithelial cells (ARPE-19, American Type Culture Collection, Manassas, VA, USA) were cultured in Dulbecco’s modified Eagle Medium/F-12 (DMEM/F-12, WELGENE Inc., Daegu, Republic of Korea) supplemented with 10% fetal bovine serum (FBS) and penicillin (60 IU/mL)/streptomycin (50 μg/mL) at 37 °C in a humidified atmosphere of 5% CO_2_.

### 4.2. Oxidative Injury of ARPE-19 Cells

Cells were grown to 70 % confluence in 96-well plates and treated with various concentrations of esculetin (Sigma Aldrich, Louis, MO, USA; 0, 10, 25, 50, 100, 250, 500, and 100 μM) and *tert*-butyl hydroperoxide (*t*-BHP, Sigma Aldrich, Louis, MO, USA; 0, 50, 100, 200, 250, 300, 350, and 400 μM) for 24 h. Cell viability was examined using a CCK cell viability assay kit (Donginbiotech Co, Seoul, Republic of Korea).

### 4.3. Measurement of Reactive Oxygen Species (ROS) Generation

The fluorescent dye 2′,7′-dichlorodihydrofluorescein diacetate (DCF-DA; Sigma Aldrich, Louis, MO, USA) and CellROX green reagent (Thermo Fisher Scientific, Inc., Waltham, MA, USA) was used to detect intracellularly active oxygen. After 24 h of treatment with *t*-BHP (300 μM) and esculetin (0, 10, 25, and 50 μM), cells were treated with 3 μM DCF-DA or 5 μM CellROX green reagent for 30 min and washed with HBSS (WELGENE Inc., Daegu, Republic of Korea). The DCF-DA-positive signal intensity was measured using a Spark^®^ Multimode Microplate Reader (Tecan, Männedorf, Switzerland). The fluorescence intensity of CellRox Green was measured using a Spark^®^ Multimode Microplate Reader following the manufacturer’s instructions. The CellROX-positive cells were also detected with a fluorescence microscope (BX51, Olympus, Tokyo, Japan).

### 4.4. TUNEL Staining

Terminal deoxynucleotidyl transferase dUTP nick-end labeling (TUNEL) staining was performed using a one-step TUNEL kit (Roche, Mannheim, Germany) according to the manufacturer’s instructions. Apoptotic cells were assessed under a fluorescence microscope at 100× magnification. In this assessment, TUNEL-positive apoptotic cells were identified using green fluorescence emitted by the cells (BX51, Olympus, Tokyo, Japan). ImageJ software (NIH, Bethesda, MD, USA) was used to count TUNEL-positive cells.

### 4.5. Apoptosis Antibody Array

The apoptosis-related signaling pathways were analyzed using Proteome Profiler™ (R&D Systems, Wiesbaden, Germany) according to the manufacturer’s instructions. Protein expression levels were determined using an image analyzer (ATTO, Tokyo, Japan).

### 4.6. Western Blot Analysis

Western blotting was used to evaluate the expression levels of apoptosis-related proteins (Bax, Bcl-2, PARP, and caspase-3) in ARPE-19 cells. The antibodies were anti-Bax, anti-Bcl2, anti-PARP, and anti-caspase-3 (Abcam, Waltham/Boston, MA, USA). Finally, a western blotting detection kit (SuperSignal™ West Dura Extended Duration Substrate, ThermoFisher Scientific, Waltham, MA, USA) was used to observe the protein bands after treatment with an HRP-conjugated secondary antibody (Advansta, San Jose, CA, USA). Protein expression levels were determined using an image analyzer (ATTO, Tokyo, Japan).

### 4.7. Real-Time PCR

Total RNA was extracted from the collected cells using the TRIzol™ reagent (Invitrogen, Carlsbad, CA, USA). The primers used were as follows: Bax, 5′-AAACTGGTGCTCAAGGCCC-3′ and 5′-CTTCAGTGACTCGGCCAGG-3′; Bcl2, 5′-GATAACGGAGGCTGGGATGC-3′ and 5′-TCACTTGTGGCCCAGATAGG-3′; Caspase-3, 5′-TTGGACTGTGGGATTGAGACG-3′ and 5′-CGCTGCACAAAGTGACTGGA-3′; PARP, 5′-GCTTCAGCCTCCTTGCTACA-3′ and 5′-TTCGCCACTTCATCCACTCC-3′; β-actin, 5′-CTCACCCTGAAGTACCCCATC-3′ and 5′-GGATAGCACAGCCTGGATAGCA-3′. PCRs were performed using a MiniOpticon™ real-time PCR system (Bio-Rad, Hercules, CA, USA) and 2xSYBR^®^ Green PCR Master Mix (Enzo, New York, NY, USA). The results were normalized to β-actin levels. The experiments were performed in triplicate, with three independent repetitions.

### 4.8. Statistical Analysis

Data are expressed as the mean ± standard error of the mean (SEM). One-way analysis of variance (ANOVA) was performed, followed by Tukey’s posthoc test using the Prism 8.0 software (GraphPad, San Diego, CA, USA).

## Data Availability

The data presented in this study are available on request from the corresponding author.

## Sample Availability

Samples of the compounds are available from the authors.
